# Exposure to Glyphosate and Chlorpyrifos Induces Oxidative Stress, Potentially Impacting Sex Determination in Zebrafish (*Danio rerio*)

**DOI:** 10.3390/jox16030101

**Published:** 2026-06-02

**Authors:** Daniela Arias-Camacho, Brian Antonio Rochin-Peraza, Miguel Betancourt-Lozano, Selene Abad-Rosales, José Basilio Heredia, Nayely Leyva-López, Samuel Calderón-Liévanos, Alejandra García-Gasca

**Affiliations:** 1 Molecular Biology and Cell Culture Laboratory, Research Centre for Food and Development, Avenida Sábalo Cerritos s/n, Mazatlán 82112, Mexico; darias226@estudiantes.ciad.mx (D.A.-C.); nrochin123@estudiantes.ciad.mx (B.A.R.-P.); 2 Ecotoxicology Laboratory, Research Centre for Food and Development, Mazatlán 82112, Mexico; mbl@ciad.mx; 3 Fish Histopathology Laboratory, Research Centre for Food and Development, Mazatlán 82112, Mexico; selene@ciad.mx; 4 Functional Food and Nutraceutical Laboratory, Research Centre for Food and Development, Carretera a El Dorado Km 5.5, Col. Campo El Diez, Culiacán 80110, Mexico; jbheredia@ciad.mx (J.B.H.); nayely.leyva@ciad.mx (N.L.-L.)

**Keywords:** DNA damage, pesticide exposure, environmental concentrations, neurotoxicity, gonadal development, endocrine disruption, vertebrates

## Abstract

The widespread use of pesticides in agriculture has increased their levels in water bodies, threatening aquatic ecosystems. Among these, glyphosate and chlorpyrifos are widely used in Mexico and can cause toxic effects even at low doses. In aquatic organisms, early exposure to these pollutants can disrupt vital processes, such as sex determination, through oxidative stress. This study assessed the effects of exposure to environmental concentrations of glyphosate (100 μg/L), chlorpyrifos (1.5 μg/L), and their combination on zebrafish (*Danio rerio*) from early stages to 90 days post fertilisation (dpf). Survival was measured using Kaplan–Meier curves; lipid peroxidation was assessed by malondialdehyde (MDA); sex-related gene expression was measured by qPCR of selected genes at 30 dpf; and gonadal development was assessed by histology at 65 dpf. The results showed increased MDA levels in exposed fish. Glyphosate caused early toxicity and a higher proportion of undifferentiated fish, implying delayed sex determination. Chlorpyrifos induced oxidative stress and affected *amh* gene expression linked to masculinisation. Combined exposure reduced survival and altered gene expression and gonadal development. Exposure shifted the sex ratio toward males, suggesting that pesticide-induced oxidative stress may alter the expression of sex determination genes during early development.

## 1. Introduction

In vertebrates, sex determination directs gonadal development towards ovaries or testes through genetic, hormonal, and cellular signals [1,2]. In juvenile zebrafish (*Danio rerio*), gonads are bipotential, with sex differentiation occurring between 20 and 30 days post fertilisation (dpf) [3,4]. Initially resembling an immature ovary, the gonads of juvenile zebrafish develop along two pathways: approximately half differentiate into ovaries, characterised by activation of cytochrome p-450 19a1a (*cyp19a1a*) and forkhead box protein L2 (*foxl2*), whereas the remaining half undergo apoptosis and differentiate into testes, involving the activation of doublesex and mab-3-related transcription factor 1 (*dmrt1*), SRY-box transcription factor 9 (*sox9a*), and anti-Müllerian hormone (*amh*) [5,6,7,8,9,10]. Apoptosis is crucial for masculinisation; juvenile male zebrafish exhibit germ cell apoptosis between 23 and 35 dpf, facilitating the ovary-to-testis transition [6]. This developmental window is particularly sensitive to environmental stressors because germ cell proliferation, apoptosis, and gonadal differentiation occur concurrently during early juvenile development [9].

Oxidative stress, resulting from excessive reactive oxygen species (ROS), can damage lipids, proteins, and DNA, thereby impairing germ cells and causing DNA damage, chromosomal abnormalities, and cell death, posing a threat to reproductive health [11,12,13,14,15,16]. Environmental pollutants, such as pesticides widely used in Mexican agriculture, can induce oxidative stress, affecting germ cells and gonadal development [17,18,19]. Pesticides frequently enter aquatic ecosystems through agricultural runoff and wastewater discharges, posing a major threat to aquatic organisms [20,21]. Approximately 64% of agricultural watersheds worldwide are considered at risk of pesticide contamination [22]. Because aquatic organisms are often exposed to multiple co-occurring contaminants, pesticide mixtures may induce additive, synergistic, or antagonistic effects that differ from those of individual compounds. Nevertheless, current ecological risk assessments continue to focus primarily on single chemicals [23].

Glyphosate and chlorpyrifos are agricultural pesticides that induce oxidative stress and cause damage in aquatic species; glyphosate affects oocytes by increasing ROS and inducing DNA damage, apoptosis, and autophagy, whereas chlorpyrifos is associated with neurotoxicity and genotoxic effects [24,25,26]. Chlorpyrifos has been frequently detected in freshwater systems from agricultural regions at concentrations ranging from 0.005 to 10.8 μg/L [27,28], while glyphosate concentrations in surface waters may range from 0.13 to 42.8 μg/L and may exceed 100 μg/L in highly impacted environments [29,30]. These findings support the environmental relevance of the concentrations used in the present study. However, most previous studies have focused on acute exposure scenarios or single compounds, whereas information regarding chronic co-exposure during the critical window of gonadal differentiation remains limited. Although oxidative stress has been proposed as an important mechanism underlying pesticide toxicity [14,31,32,33], the relationship between oxidative stress and sex differentiation-related processes in zebrafish remains poorly understood, particularly under chronic co-exposure conditions [34,35]. Therefore, this study aimed to evaluate whether glyphosate and chlorpyrifos, individually and in combination, are associated with oxidative stress responses, changes in the expression of sex differentiation-related genes, and alterations in sex ratios in zebrafish.

## 2. Materials and Methods

### 2.1. Preparation of Solutions

The experimental solutions were made using glyphosate (GLY; AccuStandard^®^, Chem Service Inc., West Chester, PA, USA) and chlorpyrifos (CPF; PESTANAL^®^, Sigma-Aldrich, St. Louis, MO, USA). The glyphosate stock solution (1.036 mg/mL) was prepared with analytical-grade distilled water, while the chlorpyrifos stock solution (14.4 μg/mL) was prepared with analytical-grade ethanol. Both solutions were diluted in reverse osmosis-treated water to achieve the concentrations used in the *D. rerio* experimental setup.

### 2.2. Zebrafish Maintenance and Embryo Production

Adult zebrafish (>6-month-old; 39.5 ± 2.8 mm in length; 0.52 ± 0.15 g in weight) were obtained from a well-established genetic line housed under controlled conditions at the Ecotoxicology Laboratory of the Centro de Investigación en Alimentación y Desarrollo (CIAD), Mazatlán, Mexico. Fish were maintained in 6 L aquaria containing reverse osmosis-treated water at 28 ± 1 °C, under a 14 h light:10 h dark photoperiod. They were fed twice daily with a commercial zebrafish diet (Zeigler, Apopka, FL, USA), supplemented with *Artemia* nauplii, following the guidelines outlined by Lawrence et al. [28].

Embryos were obtained following the established protocol in the Ecotoxicology Laboratory at CIAD. Organisms were produced by the natural spawning of one group of eight zebrafish, each bred in pairs using slope tanks. The males and females were separated overnight; the divider was removed the following day, and eggs were collected on the same day. Approximately 4 h post fertilisation (hpf), embryos were examined under a stereoscopic microscope (Stemi 305, Carl Zeiss, Oberkochen, Germany), and those at the blastula stage were selected based on the embryonic development classification described by Kimmel et al. [36]; viable embryos were selected based on synchronous development, regular cell divisions, absence of visible abnormalities, and a homogeneous appearance. All experimental procedures were approved by the Research Ethics Committee at the Centro de Investigación en Alimentación y Desarrollo (registration CONBIOETICA-26-CEI-001-20200122; approval number CEI/022-1/2022).

### 2.3. Experimental Design

A total of 800 embryos were randomly assigned to four experimental groups using the “RAND()” function in Excel: control (no exposure), glyphosate (GLY 100 μg/L), chlorpyrifos (CPF 1.5 μg/L), and a combined treatment of both pesticides (GLY 100 μg/L + CPF 1.5 μg/L). The concentrations were selected based on preliminary experiments that showed no acute mortality and permitted long-term exposure. A separate solvent control was not included, as preliminary tests conducted under controlled conditions showed no effects attributable to the ethanol vehicle. Each group consisted of five replicates, with 40 organisms per replicate (sample size was determined based on previous studies conducted in our laboratory). The experimental unit was the independent tank. Embryos were initially maintained in Petri dishes containing the respective experimental solutions until hatching. Subsequently, larvae were transferred to 2 L glass aquaria and maintained under identical conditions until 90 dpf. Exposure concentrations were selected based on reported levels in aquatic environments and preliminary assays that identified sublethal doses. Nominal concentrations were used throughout the experiment and were not analytically confirmed during exposure. 

To minimise potential confounding factors and cross-contamination between groups, replicates of each experimental group were maintained in separate containers (water baths). Animals were maintained at 28 ± 1 °C in a thermostatically controlled water bath with continuous aeration to ensure adequate water circulation and temperature stability, under 14 h light:10 h dark photoperiod. Fish were fed twice daily with finely ground commercial zebrafish feed (Zeigler, Apopka, FL, USA). Uneaten feed and waste were removed after each feeding, and 80% of the water was renewed every four days.

**Figure 1:**
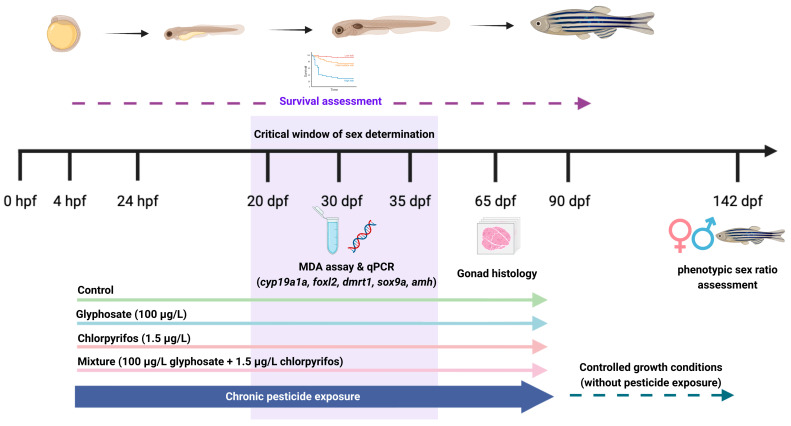
Experimental design and sampling timeline for chronic exposure of zebrafish to glyphosate, chlorpyrifos, and their combination.

Exposure was conducted from the embryonic stage until 90 dpf, encompassing the critical window for sex determination and gonadal differentiation, which occurs between 20 and 60 dpf in *D. rerio*. Two main sampling points were established during the study. At 30 dpf, a total of 20 organisms (one per replicate, *n* = 5 replicates per treatment) were collected for lipid peroxidation analysis. These organisms were individually weighed using an analytical balance (Entris^®^ II Essential Line, BCE124-1S; Sartorius, Germany) prior to biochemical analysis. Additionally, 40 organisms (two per replicate, *n* = 5 replicates per treatment) were collected for gene expression analysis. At 65 dpf, 40 organisms (two per replicate, *n* = 5 replicates per treatment) were sampled for histological evaluation of gonadal development (Figure 1). One control replicate tank (R5) was excluded from further analyses because a temperature spike during the experiment compromised the reliability of its data, leaving only four control replicates. Organisms were euthanised using an overdose of 2% lidocaine to minimise stress and prevent pain. Samples for molecular analyses were preserved in RNAlater (Thermo Fisher Scientific, Waltham, MA, USA), whereas those for histology were fixed in 4% formaldehyde. The remaining individuals not included in the present study were utilised in a parallel investigation (in accordance with the protocol approved by the Research Ethics Committee). Due to the complexity of the experiments and the number of organisms allocated to this and other studies, blinding was not feasible; therefore, all personnel were aware of group assignments throughout the experiment. 

Following the 90-day post-exposure period, 111 surviving organisms were maintained in pesticide-free water for approximately 8 days to allow for recovery. Experimental solutions were gradually replaced through water renewals every two days, reducing treatment concentrations to 80%, 50%, 20%, and finally 0%. Subsequently, zebrafish were transferred to the culture system and maintained under optimal growth conditions in 8 L aquaria with continuous water circulation. Fish were fed a commercial zebrafish diet supplemented with *Artemia* sp., and handling was minimised to reduce stress. Once organisms reached sufficient maturity for external identification, phenotypic sex was determined to assess potential changes in the sex ratio associated with early pesticide exposure. Phenotypic sex classification was based on external secondary sexual characteristics, including body shape, abdominal distension, and body colouration, in accordance with established zebrafish sex differentiation criteria [37]. Although phenotypic sexing is commonly used in adult zebrafish, it may be less precise than histological evaluation.

### 2.4. Gene Expression

Total RNA was extracted at 30 dpf using TRIzol reagent (Thermo Fisher Scientific, Waltham, MA, USA) according to the manufacturer’s instructions. Whole organisms were used for RNA extraction due to their small size at this developmental stage, as gonadal dissection could result in substantial tissue loss. RNA concentration and purity were determined using a NanoDrop™ spectrophotometer (Thermo Fisher Scientific, USA), based on absorbance at 260 and 280 nm. Subsequently, DNase digestion was performed using the TURBO DNA-free™ kit (Thermo Fisher Scientific, Waltham, MA, USA) to remove potential genomic DNA contamination.

Complementary DNA (cDNA) synthesis was performed using M-MLV Reverse Transcriptase (Promega Corporation, Madison, WI, USA) and random primers. Gene expression quantification was performed by qPCR using the Luna^®^ Universal qPCR Master Mix kit (New England Biolabs, Ipswich, MA, USA) in a CFX Opus 96 thermocycler (Bio-Rad Laboratories, Hercules, CA, USA).

The expression of sex differentiation-related genes (*cyp19a1a*, *foxl2*, *dmrt1*, *amh*, and *sox9a*) was evaluated, and *β-actin*, *rpl13a*, and *ef1α* were used as reference genes. These reference genes were selected based on Tang et al. [38], who evaluated several reference genes in zebrafish across different developmental stages. Their suitability was further confirmed through preliminary expression stability assessments and by their consistent performance in previous experiments conducted in our laboratory under similar exposure conditions. Oligonucleotide sequences are presented in Table 1. The qPCR conditions consisted of an initial denaturation of 30 s at 95 °C, followed by 40 cycles of 20 s at 95 °C, 20 s at the specific annealing temperature for each primer pair, and a 20 s extension at 60 °C. Amplification efficiencies were calculated from standard curves, and relative gene expression levels were determined using the Pfaffl (2004) method [39] (Supplementary File S1, Figures S1–S3, Table S1). Because *D. rerio* gonadal differentiation is still ongoing at 30 dpf, gene expression patterns at this stage were interpreted cautiously as indicators of sex differentiation-related pathways rather than definitive sex assignment. Previous studies have shown that genes such as *dmrt1*, *sox9a*, *amh*, *foxl2*, and *cyp19a1a* exhibit sex-biassed expression patterns during juvenile gonadal differentiation between 25 and 30 dpf, despite the absence of fully differentiated gonadal morphology [10,40,41]. Furthermore, the juvenile ovary phase in zebrafish is highly variable among individuals, and molecular differentiation may precede clear histological differentiation [42,43,44]. To explore sex differentiation-related molecular trends at 30 dpf, individuals were categorised based on the relative expression balance between male-associated genes (*dmrt1*, *amh*, and *sox9a*) and female-associated genes (*cyp19a1a* and *foxl2*), following an approach adapted from Jørgensen et al. [40]. An integrative expression balance was calculated by comparing the relative expression of male- and female-associated genes. Based on the resulting expression patterns, individuals were categorised as exhibiting male-biassed, female-biassed, intermediate differentiation-related, or undetermined expression profiles, with the latter assigned when no clear molecular pattern was observed. Because gonadal differentiation remains dynamic at this developmental stage, these classifications were interpreted as exploratory molecular profiles rather than definitive sex assignments.

### 2.5. Lipid Peroxidation

Lipid peroxidation was measured by quantifying malondialdehyde (MDA) in whole organisms at 30 dpf using a colourimetric method based on modified protocols from Lizárraga-Velázquez et al. [45] and Solé et al. [46].

For the assay, 50 μL of the supernatant obtained by macerating the experimental organisms in PBS buffer (50 mM, pH 7.4) was combined with 325 μL of 1-methyl-2-phenylindole solution (10.3 mM, in methanol/acetonitrile, 1:3; *v*/*v*), 50 μL of distilled water, and 75 μL of 37% HCl. This reaction mixture was incubated at 45 °C for 40 min. Subsequently, the reaction solution was cooled on ice for 10 min and then centrifuged at 3000× *g* for 15 min at 4 °C to remove particulate matter, yielding a clear supernatant. After centrifugation, the supernatant was transferred to a microplate, and the absorbance was recorded at 586 nm using a Synergy HT microplate spectrophotometer (BioTek Instruments, Inc., Winooski, VT, USA). Calculations were performed using a 1,1,3,3-tetramethoxypropane standard curve (Sigma Aldrich 108383-100 ML) (0 to 10 µM), and results were expressed as micromoles of malondialdehyde per gram of tissue (μmol MDA/g tissue). MDA levels are shown in Supplementary File S2. MDA was used as an indicator of lipid peroxidation and oxidative damage; however, additional antioxidant biomarkers were not assessed.

### 2.6. Gonad Histology

Fish collected on day 65 dpf were fixed in 4% formaldehyde solution, dehydrated and embedded in paraffin. Serial transverse sections (5 µm thick) spanning the abdominal cavity and gonadal region along the anterior–posterior axis were obtained using a rotary microtome (Leica Microsystems, model LEC-RM2125RTS, Leica, Wetzlar, Germany). Histological analysis was performed on 38 fish, yielding 271 serial slides (approximately seven per fish, depending on specimen size). The serial sections were examined throughout the body cavity to enable detailed evaluation of gonadal structures at multiple depths.

Histological sections were stained with haematoxylin-eosin and examined using an Olympus CX31 microscope (Tokyo, Japan) coupled with a QImaging digital camera and the QCapture Pro image acquisition software (v 6.0.0.605, 2000/xp) to determine the phenotype and sex ratio of *D. rerio* based on established histological characteristics, including the presence of perinucleolar oocytes and ovarian cavity formation in females, and the presence of spermatogonia, spermatocytes, and testicular lumen organisation in males. The presence of cellular damage and structural alterations in the gonads was also assessed in accordance with Kinnberg et al. [47] and Silva et al. [48].

### 2.7. Statistical Analysis

Statistical analyses were performed using the R environment (version 4.4.2). Survival was analysed using Kaplan–Meier curves, and differences among experimental groups were assessed using the log-rank test.

Gene expression, oxidative stress, and gonadal development data were initially evaluated for normality (Shapiro–Wilk test) and homoscedasticity (Levene’s test). When parametric assumptions were met, a one-way analysis of variance (ANOVA) was performed, followed by Tukey’s post hoc test. Otherwise, the nonparametric Kruskal–Wallis test was applied, followed by multiple comparisons using Dunn’s test. Correlation between oxidative stress and gene expression levels was assessed using Spearman’s correlation coefficient. Sex ratios were analysed using Fisher’s exact test. Statistical significance was set at *p* < 0.05.

## 3. Results

### 3.1. Survival

**Figure 2:**
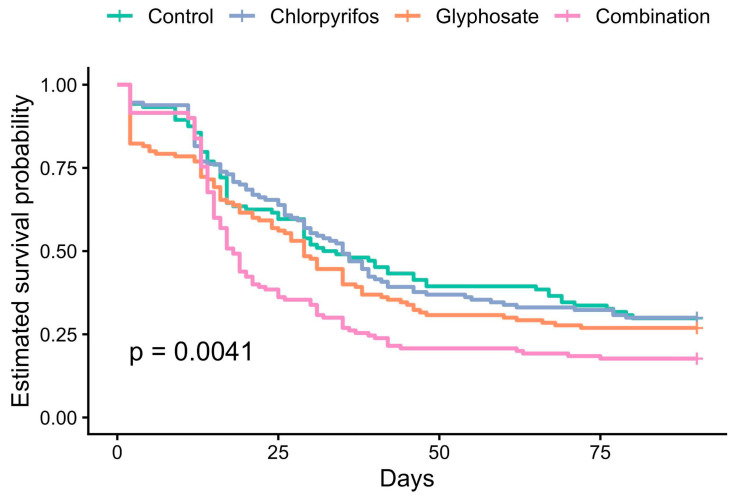
Cumulative survival of zebrafish exposed for 90 days, assessed using Kaplan–Meier method.

Survival over the 90-day exposure period was assessed using Kaplan–Meier curves. During the first 24 h, organisms exposed to glyphosate exhibited significantly higher mortality than those in the control and chlorpyrifos groups (*p* < 0.05). No significant differences were observed among the control, chlorpyrifos, and combined groups (*p* > 0.05). Over the full 90-day period, the Log-Rank test revealed significant differences among experimental groups (*p* = 0.0041). Survival in the combination group was significantly lower than in the control (*p* = 0.0108) and chlorpyrifos groups (*p* = 0.0076), while no significant difference was observed between the combination and glyphosate groups (*p* = 0.0712). Final survival was 29.8% in the control group (*n* = 160 organisms), approximately 30% in the glyphosate and chlorpyrifos groups, and 17.7% in the combined treatment group (*n* = 200 organisms per pesticide treatment). No significant differences were detected among control, chlorpyrifos, and glyphosate groups (*p* > 0.05) (Figure 2).

### 3.2. Effects of Pesticide Exposure on Lipid Peroxidation

**Figure 3:**
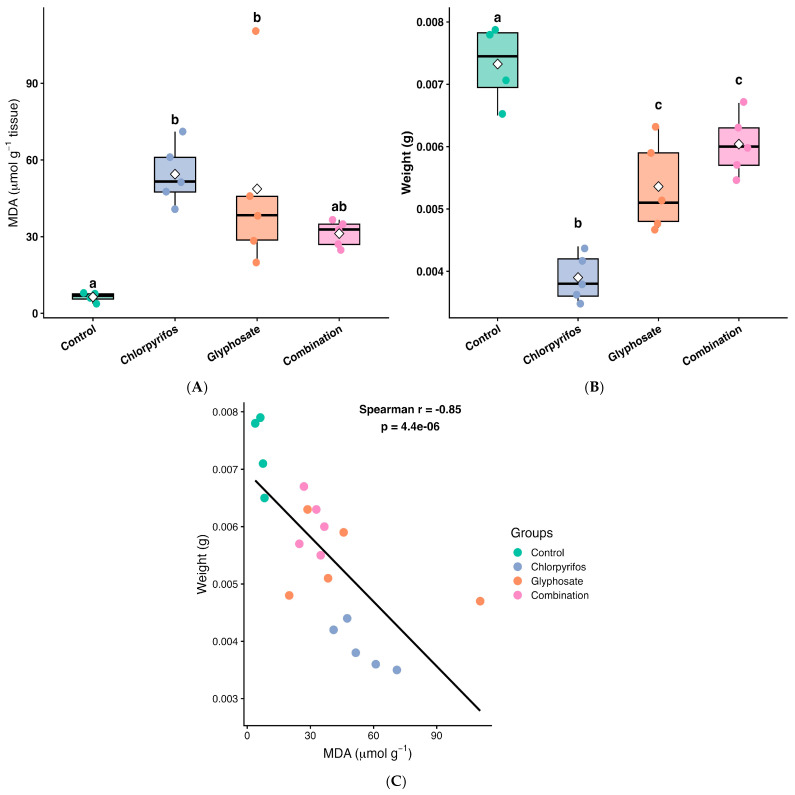
(**A**) Malondialdehyde (MDA; µmol/g tissue) concentrations in fish exposed to glyphosate, chlorpyrifos, and their combination at 30 dpf (*n* = 4 for the control group, and *n* = 5 for all pesticide treatments). (**B**) Body weight (g) distribution at 30 dpf across experimental groups. (**C**) Relationship between MDA levels and body weight (significant negative correlation; Spearman ρ = −0.85, *p* < 0.00001). Different lowercase letters indicate significant differences (*p* < 0.05). White diamonds represent mean values.

Lipid peroxidation analysis revealed significant differences among treatments (*p* = 0.0115). Organisms exposed to chlorpyrifos exhibited the highest MDA levels (54.53 ± 1.94 μmol MDA/g of tissue), significantly higher than those of the control group (6.41 ± 1.94 μmol MDA/g of tissue; *p* = 0.0119). Organisms exposed to glyphosate also showed a significant increase compared to the control (48.70 ± 36.00 μmol MDA/g of tissue; *p* = 0.0279). However, in the combined treatment group (31.20 ± 5.14 μmol MDA/g of tissue), MDA levels did not differ significantly from those observed in the chlorpyrifos (*p* = 0.2859), glyphosate (*p* = 0.5190), or control group (*p* = 0.2814). Similarly, no significant differences were observed between the glyphosate and chlorpyrifos groups (*p* = 0.9666) (Figure 3A). This increase in lipid peroxidation was associated with a significant decrease in body weight. Statistical analysis revealed significant differences in body weight (*p* = 0.0016), with fish exposed to chlorpyrifos exhibiting lower values (0.00390 ± 0.000387 g) compared to the control group (0.00732 ± 0.000655 g) (Figure 3B). Furthermore, a strong negative correlation was observed between MDA levels and body weight (Spearman ρ = −0.85, *p* < 0.00001) (Figure 3C).

### 3.3. Effects of Pesticide Exposure on Gene Expression 

Mean RNA concentration was 169.4 ng/µL. Average purity ratios (A260/280) were 1.86, indicating acceptable RNA quality for downstream applications.

**Figure 4:**
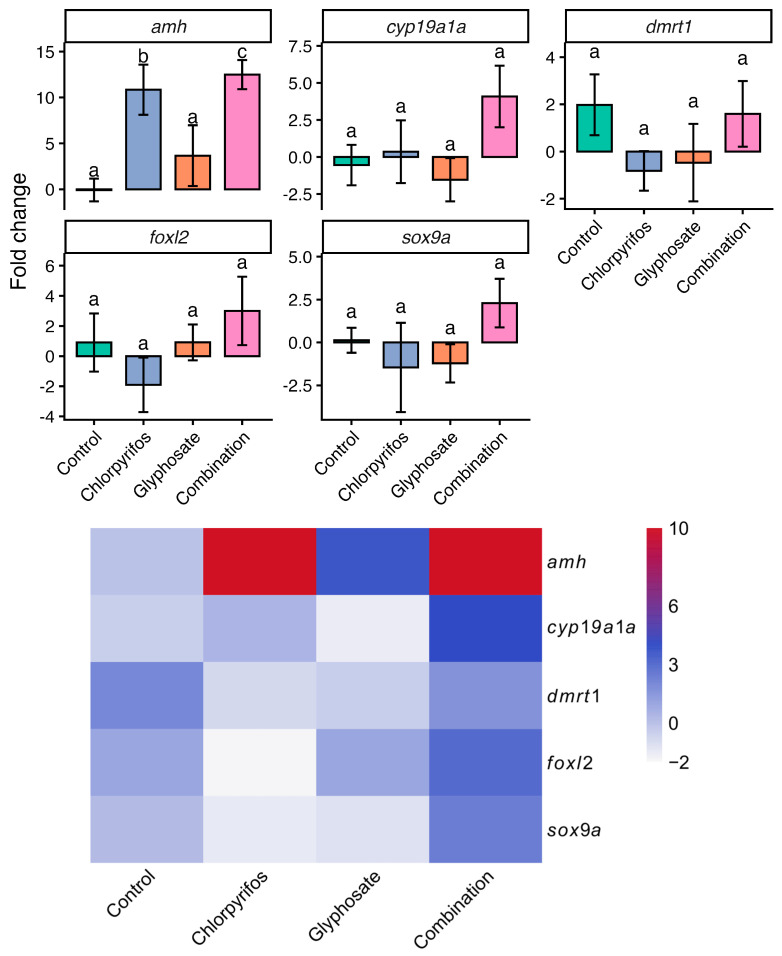
**Top**: Relative expression of sex-related genes (*amh*, *cyp19a1a*, *dmrt1*, *foxl2*, and *sox9a*) in zebrafish exposed to glyphosate, chlorpyrifos, and their combination at 30 dpf (*n* = 4 for the control group, and *n* = 5 for all pesticide treatments). **Bottom**: Overall expression patterns of sex-determination genes across experimental groups. Different lowercase letters indicate significant differences (*p* < 0.05).

Gene expression of sex determination-related markers, including *dmrt1*, *amh*, *sox9a*, *cyp19a1a*, and *foxl2*, was analysed. Among these, only *amh* showed a significant difference among groups (*p* = 0.0021) (Figure 3). Fish exposed to chlorpyrifos and the pesticide mixture exhibited significantly higher *amh* expression compared to the control group (*p* = 0.0036). In contrast, the expression levels of *dmrt1*, *sox9a*, *cyp19a1a*, and *foxl2* did not differ significantly among groups (*p* > 0.05). Gene expression was quantified as log2 fold change (log2FC) relative to the control group, where values represent normalised fold changes on a logarithmic scale (FC = 2^log2FC; positive values indicate upregulation and negative values indicate downregulation). In control organisms, expression levels were centred around baseline values (*dmrt1*: 0.00 ± 5.53; *amh*: 0.00 ± 3.30; *sox9a*: 0.00 ± 1.83; *cyp19a1a*: 0.00 ± 3.87; *foxl2*: −0.73 ± 4.04), reflecting normalised reference conditions. Chlorpyrifos exposure induced strong upregulation of *amh* (10.84 ± 6.11), together with downregulation of *dmrt1* (−0.82 ± 2.65) and *sox9a* (−1.46 ± 4.51), while *cyp19a1a* (0.36 ± 6.36) and *foxl2* (−1.91 ± 5.11) showed variable responses. Glyphosate exposure resulted in moderate *amh* upregulation (3.66 ± 6.63) and general downregulation of *dmrt1* (−0.47 ± 5.20) and *sox9a* (−1.21 ± 2.95), while *cyp19a1a* (−1.54 ± 3.26) and *foxl2* (0.91 ± 1.68) showed minor variation. In contrast, the combination treatment showed the strongest transcriptional response, with marked upregulation of *amh* (12.49 ± 4.77), followed by *sox9a* (2.29 ± 4.25), *cyp19a1a* (4.08 ± 5.09), *foxl2* (3.00 ± 6.00), and *dmrt1* (1.59 ± 4.40). In fish exposed to glyphosate, relative expression levels of the analysed genes tended to decrease, although overall, gene expression tended to increase when combined with chlorpyrifos (Figure 4).

### 3.4. Correlation Analysis Among Sex Determination Genes and Lipid Peroxidation (MDA) Levels

**Figure 5:**
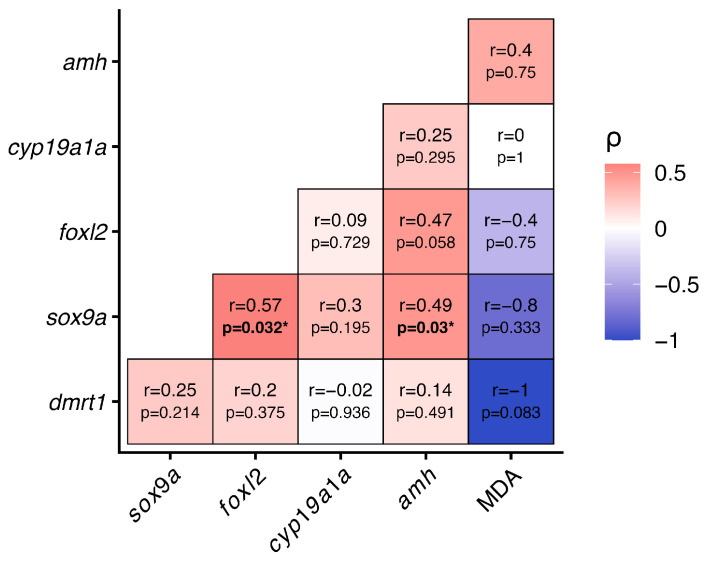
Correlation analysis of sex determination genes and lipid peroxidation (MDA) in zebrafish exposed to glyphosate, chlorpyrifos, and their combination at 30 dpf. Asterisks (*) indicate significant positive correlation based on Spearman’s test.

Correlation analysis among genes involved in sex determination revealed a significant positive result between *amh* and *sox9a* (r = 0.568, *p* = 0.015). Similarly, a significant positive correlation was found between *sox9a* and *foxl2* (r = 0.551, *p* = 0.041). (Figure 4). The relationship between MDA levels and the expression of *dmrt1*, *amh*, *sox9a*, *cyp19a1a*, and *foxl2* was also analysed. No statistically significant correlations were found (*p* > 0.05) (Figure 5).

### 3.5. Effects of Pesticide Exposure on Sex Ratios at 30 dpf

To explore the potential effects of treatments on sex determination, individuals were classified based on their gene expression profiles using an index that integrates the relative expression of genes associated with male (*dmrt1*, *amh*, *sox9a*) and female (*cyp19a1a*, *foxl2*) phenotypes. Based on this index, fish were categorised as male-biassed, female-biassed, intermediate, or indeterminate according to the predominance of male- or female-associated expression profiles. Intermediate individuals exhibited mixed expression profiles of male- and female-associated genes, whereas indeterminate individuals lacked a clear or consistent gene expression pattern.

**Figure 6:**
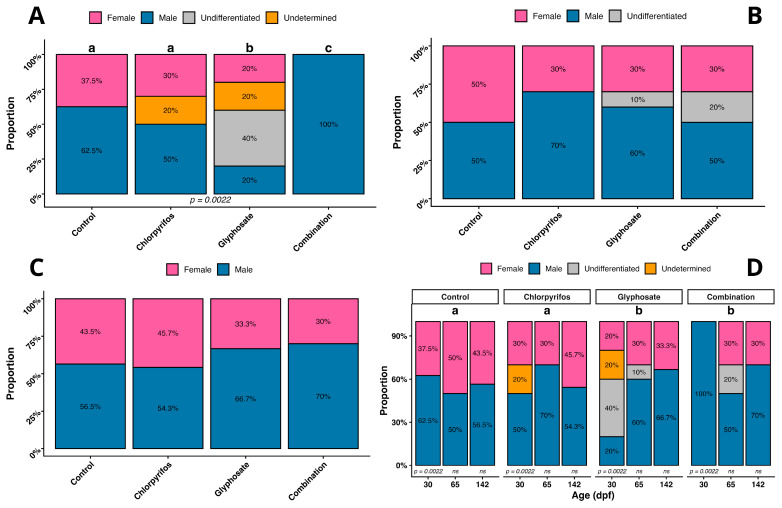
Sex ratio classification of zebrafish exposed to glyphosate, chlorpyrifos, and their combination across developmental stages. (**A**) Classification based on gene expression at 30 dpf. (**B**) Classification based on histological analysis at 65 dpf, and (**C**) sex classification based on phenotypic identification at 142 dpf. (**D**) Temporal comparison of expression profile distributions and sex classifications across developmental stages. Different lowercase letters indicate significant differences (*p* < 0.05); ns indicates non-significant differences.

In the control group, 37% (3/8) exhibited male-biassed expression profiles, whereas 63% (5/8) exhibited female-biassed expression profiles. In the chlorpyrifos treatment, the distribution was 50% male-biassed (5/10), 30% female-biassed (3/10), and 20% intermediate (2/10). Organisms exposed to glyphosate showed greater variability, with 20% male-biassed (2/10), 20% female-biassed (2/10), 20% intermediate (2/10), and 40% indeterminate (4/10). In contrast, in the combined pesticide treatment, all organisms exhibited male-biassed expression profiles (10/10) (Figure 6A).

Statistical analysis revealed significant differences in expression profile distributions among experimental groups (*p* = 0.0022) (Figure 6A). Pairwise comparisons indicated significant differences between the combined treatment and the control group, as well as between the glyphosate and chlorpyrifos treatments (*p* < 0.05). Additionally, a significant difference was observed between the control group and the glyphosate treatment. No significant differences were found between the control and the chlorpyrifos groups, nor between the glyphosate and chlorpyrifos treatments (*p* > 0.05).

### 3.6. Effects of Pesticide Exposure on Sex Ratios at 65 dpf

Fish were classified as male, female, or undifferentiated based on histological examination of the gonads (Figure 6B). The sex ratio in the control group was 50% males and 50% females (*n* = 4 replicates, 2 fish per replicate). In the chlorpyrifos treatment group, 70% were males, and 30% were females (*n* = 5 replicates, 2 fish per replicate). In the glyphosate treatment group, 60% were males, 30% females, and 10% were undifferentiated (*n* = 5 replicates, 2 fish per replicate). In the combined treatment group, 50% were males, 30% were females, and 20% were undifferentiated (*n* = 5 replicates, 2 fish per replicate). No significant differences were detected among treatments (*p* = 0.7834) (Figure 6B). Histological analysis revealed no evident structural alterations in the gonads; however, undifferentiated individuals were observed in glyphosate and combination groups.

### 3.7. Sex Ratios in Exposed Zebrafish After a Recovery Period

At the end of the experimental period (90 dpf), fish underwent a recovery and growth phase during which pesticide exposure was gradually discontinued. Subsequently, organisms were transferred to the zebrafish culture system and maintained under optimal growth conditions.

At 142 dpf, sex ratios were determined through manual individual identification to assess the distribution of males and females across groups. No significant differences in sex ratios were observed among groups (*p* = 0.6031) (Figure 6C). Phenotypic sex classification included 111 organisms in total (control: *n* = 23; chlorpyrifos: *n* = 35; glyphosate: *n* = 33; combination: *n* = 20) and was based on external secondary sexual characteristics.

### 3.8. Comparative Analysis of Sex Ratios Between Treatments and Developmental Stages

Temporal analysis within experimental groups showed that sex ratios in the control and chlorpyrifos groups did not vary significantly over time (*p* > 0.05). In contrast, organisms exposed to glyphosate (*p* = 0.00058) and the combined treatment (*p* = 0.03999) exhibited significant changes over time, with increased frequencies of male-biassed expression profiles at 30 dpf followed by male-associated classifications at later developmental stages (Figure 6D).

## 4. Discussion

This study assessed the individual and combined effects of glyphosate and chlorpyrifos on zebrafish, focusing on the induction of oxidative stress and its relationship with changes in sex determination and differentiation. To this end, the following endpoints were evaluated: survival; lipid peroxidation levels as a marker of redox imbalance; classification of individuals based on gene expression profiles; histological examination of the gonads; and sex ratios among surviving organisms.

Early mortality observed in embryos within the first 24 hpf is consistent with previous studies conducted under controlled conditions. High embryo mortality during the first 10–15 h is considered a natural process that eliminates embryos with developmental abnormalities, such as asynchronous cell division [49,50]. Therefore, mortality observed in the control group may reflect spontaneous embryonic loss, which is commonly reported in this species.

In fish exposed to glyphosate, increased mortality within the first 24 h and reduced survival at 10 dpf are consistent with the compound’s high embryonic sensitivity. Previous studies have documented teratogenic effects, increased early mortality, and reduced survival following glyphosate exposure as early as 6 hpf in *D. rerio* [51,52]. Additionally, glyphosate has been shown to induce acute toxicity during early developmental stages, characterised by delayed hatching, cardiotoxicity, and morphological abnormalities [33,53,54,55]. Collectively, this evidence suggests that glyphosate may induce acute embryonic toxicity, likely mediated by the production of reactive oxygen species (ROS) and the disruption of cardiovascular development.

During the juvenile stage (30–90 dpf), survival rates in the control group (29.8%) and fish exposed to glyphosate and chlorpyrifos (26.9–30%) were slightly below the range reported under laboratory conditions (35–76%) [56], suggesting generally comparable survival under the present experimental conditions and mortality consistent with species-specific expectations. In this study, survival in the chlorpyrifos treatment group was comparable to that observed in the glyphosate group, suggesting that the concentrations used may not have significantly affected long-term survival and could have triggered compensatory physiological responses to prolonged exposure. In contrast, the combined exposure to glyphosate and chlorpyrifos resulted in the lowest survival rate (17.7%), with increased mortality beginning at 15 dpf, a stage at which natural mortality in *D. rerio* typically declines [56]. This pattern suggests a progressive reduction in physiological capacity in organisms exposed to the pesticide combination.

The interaction between glyphosate and chlorpyrifos has been documented, with effects ranging from additive to antagonistic, depending on the species, concentrations, and experimental conditions. Zhang et al. [57] reported a synergistic effect in *Cyprinus carpio* exposed to 3.5 mg/L glyphosate and 25 µg/L chlorpyrifos, resulting in significantly higher toxicity than that observed with either compound alone. This interaction may explain the lower survival rate observed under combined exposure in the present study. However, Falfushynska et al. [58] reported an antagonistic interaction in *D. rerio* exposed to a mixture of chlorpyrifos (0.1 and 3 µg/L) and Roundup (15 and 500 µg/L) over 14 days, where chlorpyrifos was identified as the primary driver of toxicity, and overall mortality was reduced under combined exposure.

The progressive decline in fish survival following combination exposure after 15 dpf suggests cumulative effects. Falfushynska et al. [58] also identified chlorpyrifos as the main contributor to oxidative stress and DNA damage, with these effects persisting even in the presence of glyphosate. This indicates that chlorpyrifos may be the primary driver of toxicity in the tested combinations, potentially disrupting essential metabolic functions. Additionally, Stevens et al. [59] reported that glyphosate can exhibit increased persistence and toxicity when interacting with other chemicals, supporting the hypothesis that the combination may lead to cumulative or interactive toxicity mechanisms that impair long-term survival. Although pure glyphosate was used in this study, commercial formulations used in real-world environments typically contain surfactants that enhance toxicity by facilitating cellular uptake [60,61,62]. Furthermore, interactions with other pesticides or co-formulants may exacerbate these effects, particularly under chronic or combined exposure scenarios. Comparisons with previous studies are limited by differences in experimental design, including exposure concentrations, duration, developmental stages, and pesticide formulations or species used. Accordingly, direct quantitative comparisons should be interpreted with caution, and only general trends in toxicological responses can be reliably inferred.

Overall, the results indicate that combined exposure to glyphosate and chlorpyrifos may reduce *D. rerio* survival through cumulative or interactive mechanisms, likely associated with oxidative stress and cellular damage. The early sensitivity to glyphosate, together with the progressive decline observed under combined exposure, suggests that the two compounds may interact additively or antagonistically depending on exposure timing and concentration, thereby impairing physiological functions during crucial developmental stages. These findings are consistent with previous studies demonstrating that pesticides can induce oxidative stress, disrupt neurodevelopment, and impair endocrine function, ultimately reducing survival and reproductive success in aquatic organisms [52,55,63]. In this context, the results highlight the importance of considering chronic and combined effects in ecotoxicological risk assessments, particularly at environmental concentrations.

The induction of oxidative stress was confirmed through lipid peroxidation analysis, a well-established marker of oxidative damage to cellular lipids. Fish exposed to chlorpyrifos exhibited a significant increase in MDA levels, approximately 8.5-fold higher than in the control group. This finding is consistent with studies identifying chlorpyrifos as a potent inducer of oxidative stress in fish [14,58,64,65,66]. These studies have reported the accumulation of oxidative lesions, elevated MDA levels, and reduced antioxidant capacity in species such as *D. rerio* and *C. carpio*, confirming its role in oxidative stress induction.

In individuals exposed to glyphosate, MDA levels increased significantly, approximately 7.6-fold over the control, indicating substantial oxidative damage that may compromise membrane integrity. This effect has been widely reported in previous studies, where glyphosate induces ROS production, redox imbalance, lipid peroxidation, and mitochondrial dysfunction [52,55,63]. The observed damage suggests that *D. rerio* is highly sensitive to this compound during early development.

A key finding was the strong negative correlation between MDA levels and body weight (Spearman’s *p* = −0.85), indicating that increased lipid peroxidation may be associated with reduced body weight. This suggests that oxidative stress may affect not only embryonic development but also somatic growth. Previous studies [31,67] have reported reductions in length and weight in fish exposed to chlorpyrifos under both short-term (96 h) and subchronic (5 and 11 days) exposure conditions. Similar relationships between lipid peroxidation and growth impairment have been reported for other contaminants, such as tributyltin [68], supporting the hypothesis that oxidative damage disrupts membrane integrity, enzyme activity, and energy metabolism, ultimately leading to growth retardation. The relationship between oxidative stress and altered gene expression observed in this study is correlational and does not establish causality. Thus, oxidative stress cannot be inferred to directly drive changes in sex-related gene expression; instead, both responses may co-occur following pesticide exposure. Because only MDA levels were measured, interpretations regarding oxidative stress pathways should be made with caution. Lipid peroxidation reflects only one aspect of redox homeostasis, and the absence of additional antioxidant biomarkers such as superoxide dismutase (SOD), catalase (CAT), and glutathione peroxidase (GPx) limits the comprehensive characterisation of antioxidant responses and the determination of whether compensatory mechanisms were activated.

Gene expression analysis allowed for the evaluation of the effects of glyphosate, chlorpyrifos, and their combination on sex determination in zebrafish. The results revealed alterations in both ovarian and testicular gene expression, indicating that exposure to these compounds may induce endocrine disruption and shift the balance between male and female developmental pathways. Although gene expression profiling was used as an indicator of sex differentiation status, histological analysis remains a more reliable and direct method for sex identification, particularly during early developmental stages such as 30 dpf, when gonadal differentiation is still ongoing. Therefore, molecular-based classification at this stage should be interpreted as a proxy rather than a definitive determination of phenotypic sex.

Overall, exposure to chlorpyrifos and the combined treatment significantly increased *amh* expression in juvenile zebrafish. In chlorpyrifos-exposed fish, *amh* expression was variable, detected only in a subset of individuals, yet those individuals exhibited high expression levels. The remaining genes analysed did not show statistically significant differences, although some variability was observed. This pattern suggests partial activation of the testicular pathway and possible modulation of ovarian signalling, consistent with the male-biassed sex ratios observed in exposed groups.

The *dmrt1*, *amh*, and *sox9a* genes play key roles in testicular differentiation and serve as markers of the male developmental pathway. Their activation can be triggered by exogenous androgens, hormonal imbalance, or stress-related stimuli. For instance, exposure of *D. rerio* to tebuconazole, a triazole fungicide, has been shown to reduce 17β-oestradiol levels, alter the E2,testosterone ratio, and inhibit *cyp19a1a*, acting as an anti-estrogenic and masculinising agent [69]. Similarly, Xie et al. [70] reported that nandrolone exposure in *D. rerio* promotes masculinisation by increasing testosterone, reducing oestradiol, inhibiting oocyte development, and activating the mitogen-activated protein kinase (MAPK) pathway, which regulates cell proliferation, differentiation, and stress response, ultimately leading to increased *dmrt1* and *amh* expression.

In this study, *amh* expression increased significantly in fish exposed to chlorpyrifos and was further elevated under combined exposure to chlorpyrifos and glyphosate, suggesting stress-associated transcriptional reprogramming rather than direct deterministic sex reversal. This finding is consistent with reports indicating that oxidative stress or endocrine disruptor compounds can induce male-biassed phenotypes through activation of the *sox9a-amh* pathway [71,72]. The positive correlation between *amh* and *sox9a* (r = 0.568, *p* = 0.015) supports their coordinated role, as *sox9a* is involved in Sertoli cell differentiation [40,73]. Although *sox9a* did not show significant changes, its increasing trend with *amh* under combined exposure suggests coordinated activation of the testicular pathway.

The expression of *dmrt1* remained stable across experimental groups, suggesting that it was not sufficient to alter its baseline expression. However, this stability may also reflect the gene’s temporal expression pattern, as *dmrt1* exhibits transient peaks between 10 and 16 dpf, followed by a decline [40,74]. Because molecular analyses were conducted at 30 dpf, *dmrt1* was likely assessed outside its critical regulatory window, potentially explaining the lack of detectable changes, assuming early exposure effects occurred. This interpretation is supported by recent findings from Augstenová & Ma [75], which emphasise that *dmrt1* plays a primary role during early sex determination. Overall, the expression patterns of *dmrt1*, *sox9a*, and *amh* suggest that masculinisation in this study was primarily mediated through Sertoli cell signalling, a mechanism also reported in studies involving endocrine-disrupting compounds (EDCs) [74,76].

The ovarian pathway is primarily regulated by *cyp19a1a*, which catalyses the conversion of androgens into oestrogens, and by *foxl2*, a transcription factor essential for maintaining ovarian identity [77,78]. In glyphosate-exposed individuals, *cyp19a1a* expression decreased, whereas a relative increase was observed under combined exposure. This pattern is consistent with evidence that glyphosate may act as an endocrine disruptor by inhibiting aromatase activity and oestrogen synthesis [79,80], potentially promoting masculinisation.

The increase in *foxl2* expression observed under combined exposure may represent a compensatory response to activation of the testicular pathway, a phenomenon commonly reported under conditions of endocrine disruptors and oxidative stress [81,82]. Oestrogenic compounds such as bisphenol A (BPA), 17α-ethinylestradiol (EE2), and hexafluoropropylene oxide trimer acid (HFPO-TA) promote feminisation by upregulating *cyp19a1a* and *foxl2* [83,84,85]. In contrast, oxidative stress tends to suppress this pathway. For example, Saputra et al. [82] demonstrated that H_2_O_2_ exposure significantly reduces the expression of *cyp19a1a*, *cyp19a1b*, and oestrogen receptors (*esr1*, *esr2α*, *esr2β*) in *D. rerio*, establishing a direct link between oxidative stress and impaired oestrogen signalling. This pattern is consistent with the present findings, where despite an initial increase in *foxl2* expression in some individuals, likely as a compensatory response, an overall reduction in *cyp19a1a* and *foxl2* expression was observed, particularly under combined exposure, suggesting a shift toward masculinisation.

In this study, a positive correlation was observed between the expression of *sox9a* and *foxl2* (r = 0.551, *p* = 0.041), which was unexpected given that these genes are associated with opposing developmental pathways. This pattern may reflect compensatory dysregulation induced by exposure-related stress, a phenomenon documented in which pollutant exposure leads to mixed or persistent sex gene expression profiles [86]. For example, combined exposure to microplastics and copper in zebrafish males has been shown to induce simultaneous overexpression of androgen and oestrogen receptors, while HFPO-TA affects genes associated with both sexes over extended periods [34,85]. The control group exhibited a balanced sex ratio, consistent with natural populations [87] and with previous studies on sex determination under experimental conditions [85,88,89].

Fish exposed to chlorpyrifos exhibited a gene expression pattern consistent with a bias toward activation of the testicular pathway and suppression of ovarian signalling, suggesting potential disruption of ovarian developmental stability rather than a complete override of sex determination processes. This pattern is consistent with transcriptomic studies showing that 31-day-old female *D. rerio* exposed to high-temperature and high-density conditions developed a male-like gene expression profile, characterised by increased *amh* and *dmrt1* expression and decreased *cyp19a1a* and *foxl2* expression [73]. These findings support evidence that stress and endocrine-disruptor compounds can induce transcriptional reprogramming, leading to masculinisation and suppression of the ovarian pathway [70,71]. Overall, these results suggest that chlorpyrifos directly influences sex determination, promoting masculinisation.

In contrast, glyphosate exposure led to a high proportion of undifferentiated individuals, suggesting delayed or disrupted sex determination. This observation is consistent with previous studies reporting that glyphosate can delay or interfere with sex differentiation, likely through oxidative stress and cellular damage in oocytes or gonadal somatic cells [25,72]. In mammals, Yahfoufi et al. [25] reported that glyphosate damages oocytes through ROS production and zinc depletion, while Zhang et al. [24] demonstrated severe effects in metaphase II oocytes, including disruption of meiotic arrest, spindle disorganisation, reduced mitochondrial membrane potential, DNA damage, increased oxidative stress, and activation of apoptosis and autophagy. Furthermore, Zhang et al. [90] reported embryonic malformations in *D. rerio*, along with alterations in developmental and apoptosis-related genes. These mechanisms may explain the undifferentiated phenotypes observed in this study, where oxidative stress likely disrupts early developmental processes prior to sex differentiation.

Fish exposed to the combined pesticide treatment exhibited pronounced masculinisation and potential impairment of ovarian development, indicating interactive toxicological effects consistent with endocrine disruption under mixed-exposure conditions. Overall, the toxicological behaviour of the glyphosate–chlorpyrifos mixture remains difficult to predict due to context-dependent interactions, including synergistic, additive, or antagonistic effects depending on developmental stage, concentration, and exposure duration. These results highlight the complexity of mixture toxicology and reinforce the need for caution when extrapolating single-compound toxicity to environmental scenarios involving multiple contaminants. This outcome reflects a complex interaction between the two compounds, similar to findings reported by Santos et al. [35] and Luzio et al. [91], who demonstrated that combinations of endocrine-disrupting compounds (e.g., synthetic oestrogens and aromatase inhibitors) can produce unexpected synergistic effects, including complete masculinisation or the development of intersex phenotypes, depending on dose and timing of exposure.

Luzio et al. [91] showed that combined exposure to 17α-ethinylestradiol (EE2) and an aromatase inhibitor in *D. rerio* results in complete masculinisation, even at concentrations where individual compounds have no effect, by disrupting the androgen–oestrogen balance. A similar mechanism may underlie the present findings: glyphosate may alter gonadal cell function and interactions, while chlorpyrifos activates the *sox9a*–*amh* pathway, leading to enhanced masculinisation under combined exposure. These results suggest that, although chlorpyrifos primarily drives activation of the male pathway, glyphosate increases gonadal susceptibility. Its early effects on oxidative stress and oocyte development may create conditions that facilitate chlorpyrifos-induced masculinisation. Exposure to endocrine-disrupting chemicals during critical windows of gonadal development can result in long-lasting or permanent alterations in sex ratios [25,92,93].

Oxidative stress has been identified as a key factor influencing sex determination in fish, primarily by affecting oocyte viability and function, which are essential for maintaining the ovarian pathway [78]. In teleost fish, direct exposure to ROS such as H_2_O_2_ can induce masculinisation without altering cortisol levels by activating testicular genes, including gonadal somatic cell-derived factor (*gsdf*), and suppressing *cyp19a1a* [71]. Similarly, pro-oxidant compounds such as chlorpyrifos, tributyltin (TBT), and glyphosate increase ROS production, induce apoptosis, and alter oocyte and follicular dynamics [68,94,95].

Correlation analysis between MDA levels and sex-determining gene expression revealed no statistically significant associations. However, *amh* was the only gene showing a positive correlation coefficient, whereas *dmrt1*, *sox9a*, *cyp19a1a*, and *foxl2* exhibited negative correlations. These findings suggest that oxidative stress may exert gene-specific and stage-dependent effects rather than a uniform regulatory mechanism, particularly impacting oocyte stability and modulating *amh* expression. This pattern is consistent with previous studies linking elevated oxidative stress to masculinisation, oocyte dysfunction, and endocrine disruption, supporting the hypothesis that oxidative stress is a key factor underlying the observed alterations in sex determination. Long-term exposure to triclosan in zebrafish has been shown to induce a testicular expression profile characterised by upregulation of *dmrt1a*, *sox9a*, and *amh*, along with repression of aromatase activity and steroid imbalance [96]. Similarly, contaminants such as tributyltin increase MDA levels and disrupt oocyte maturation in *D. rerio*, reducing the number of mature oocytes and promoting conditions that favour masculinisation [95].

The link between oxidative stress and loss of ovarian signalling is well established. In mammals, ROS, including H_2_O_2_, O_2_^−^, and HOCl, accelerate oocyte ageing, leading to microtubule disorganisation, depletion of cortical granules, and deterioration of the zona pellucida [97]. In fish, maintaining oocyte integrity is essential for preserving the ovarian pathway; disruption of this pathway can result in masculinisation, even in individuals initially developing as females [78]. The observed downregulation of *cyp19a1a* and *foxl2*, together with increased lipid peroxidation, is consistent with this mechanism, as both genes are critical for ovarian differentiation [77]. We emphasise that the associations observed between oxidative stress, gene expression, and developmental outcomes are correlational and do not demonstrate causality. Therefore, oxidative stress and sex-related changes in gene expression should be interpreted as concurrent responses to pesticide exposure rather than as indicating a direct mechanistic link.

Gonadal differentiation in *D. rerio* can extend beyond 60 dpf under certain environmental conditions, which may explain the presence of undifferentiated individuals in the studied groups. Under standard conditions, this process is typically completed between 55 and 65 dpf. Therefore, molecular classification at 30 dpf should be interpreted with caution, as sex differentiation is still ongoing and transcriptional profiles may reflect dynamic developmental states rather than a fixed sex identity. Previous studies indicate that factors such as suboptimal temperatures, synthetic oestrogens, pesticides, and polymers can delay or disrupt gonadal development [85,87,89]. In the present study, undifferentiated gonads were observed in the glyphosate and combined exposure groups, indicating that these compounds, individually or in combination, can interfere with normal gonadal differentiation. This finding is particularly relevant, as even under controlled conditions, exposure to low temperatures or xenoestrogens such as EE2 can maintain gonads in an undifferentiated state up to 60 dpf [87]. Similarly, exposure to HFPO-TA has been shown to significantly affect gonadal differentiation depending on the timing of exposure, with critical effects occurring between 21 and 42 dpf [85], highlighting the sensitivity of specific developmental windows.

Overall, the results demonstrate that the evaluated contaminants can alter the trajectory of sex determination and gonadal development in *D. rerio*. Although no statistically significant differences in sex ratios were detected, the presence of undifferentiated individuals, the masculinisation observed in certain treatments, and the histological variability indicate biologically relevant effects of exposure. This is consistent with findings by Santos et al. [35] and Dong et al. [85], which emphasise that biologically meaningful effects may occur even in the absence of statistical significance, particularly in sensitive processes such as sex determination.

Comparative analysis further indicates that the effects of these compounds on sex determination are most pronounced during early development and tend to diminish over time. An integrated evaluation across developmental stages (30, 65, and 142 dpf) revealed stage-specific responses, with the early juvenile phase being particularly susceptible. Significant differences at 30 dpf (*p* = 0.0022) indicate that this stage is especially vulnerable to glyphosate exposure and its combination with chlorpyrifos. This observation aligns with the ovarian-to-testicular transition phase described in *D. rerio* [43] and with studies showing that even minor disruptions in apoptosis, cell proliferation, and oestrogen signalling, particularly those affecting early oocyte maintenance, can trigger early masculinisation [85,87,98].

At 65 dpf, no statistically significant differences were observed among the experimental groups; however, a trend toward masculinisation persisted in organisms exposed to glyphosate and in those exposed to the combined treatment. This pattern suggests a moderate effect of pesticide exposure, insufficient to produce detectable population-level differences, yet capable of inducing subtle alterations in gonadal development. This observation is consistent with studies in *D. rerio* showing that sexual differentiation can be delayed under suboptimal environmental conditions or in the presence of contaminants, resulting in undifferentiated individuals or transitional gonadal states beyond 60 dpf [87,98]. The presence of undifferentiated individuals and histological variability at this stage supports the hypothesis of altered or prolonged gonadal differentiation.

At 142 dpf, following a recovery period of less than 50 days, no statistically significant differences were detected among groups. Nevertheless, organisms exposed to the combined treatment exhibited the highest proportion of males. This suggests that although early exposure effects may transiently influence sexual differentiation, the gonadal system tends to stabilise over time. Furthermore, the absence of exposure during this period likely contributed to the observed recovery. However, the persistence of masculinising trends in the glyphosate and combined treatments indicates that early exposure may have lasting effects, even when they are no longer statistically significant.

This observation is consistent with reports of chronic or cumulative exposure to endocrine-disrupting compounds that induce partial masculinisation without significantly altering overall sex ratios [85,89]. In the present study, fish were exposed for 90 days, and the final assessment was conducted at 142 dpf following a recovery phase without exposure. The residual masculinisation observed, therefore, suggests persistent effects beyond the exposure period. This finding is supported by evidence that androgenic compounds such as 17β-trenbolone and prochloraz can induce long-lasting or irreversible masculinising effects in *D. rerio*, highlighting that disruptions during early developmental stages may have enduring consequences even after removal of the xenobiotic [35,93].

Treatment-specific analysis further supports this interpretation. The control group remained stable over time (*p* = 1), indicating a typical and undisturbed process of sexual differentiation. Similarly, the chlorpyrifos-exposed group did not show significant temporal variation, consistent with evidence that organophosphates often do not directly induce sex biases but instead cause physiological stress, developmental delays, or increased susceptibility to environmental stressors. In contrast, groups exposed to glyphosate and the combined treatment exhibited significant temporal changes, suggesting that glyphosate, alone or in combination with chlorpyrifos, can disrupt key mechanisms involved in sex determination, including aromatase activity, oocyte development, and gonadal tissue stability [25,85,89]. These findings are consistent with studies showing that oxidative stress, germ cell apoptosis, and inhibition of estrogenic signalling by endocrine-disrupting compounds can promote masculinisation [35].

Although this study provides evidence of the effects of glyphosate, chlorpyrifos, and their combination on sex determination in *D. rerio*, the findings should be interpreted in light of the experimental design. Limitations such as small-animal and sample sizes, and the lack of additional intermediate sampling points, restrict the ability to fully resolve interactions among oxidative stress, gene expression, and histological changes. Nevertheless, the consistent patterns observed highlight the need for further research to validate and better elucidate the mechanisms underlying these effects.

## 5. Conclusions

Exposure to chlorpyrifos, glyphosate, and their combination may contribute to alterations in sex determination in zebrafish, with oxidative stress potentially serving as an underlying mechanism. Increased lipid peroxidation suggests that oxidative damage is linked to impaired growth and is likely to be associated with dysregulation of key genes involved in sexual differentiation. Chlorpyrifos appears to drive oxidative stress, potentially promoting masculinisation through upregulation of *amh*, whereas glyphosate is associated with an increased proportion of undifferentiated individuals, suggesting delayed or disrupted gonadal differentiation. Under combined exposure, these compounds show interactive effects that reduce survival and likely increase masculinization, indicating a complex toxicological interaction. Overall, the results suggest that oxidative stress-induced reprogramming may favour activation of the *sox9a*–*amh* pathway while disrupting ovarian signalling, including *cyp19a1a* and *foxl2*. Although these findings should be interpreted with caution, they suggest that even at environmental concentrations, these pesticides may disrupt essential developmental processes by acting as endocrine disruptors that influence gonadal development and sex determination in aquatic organisms.

## Figures and Tables

**Table 1:** Oligonucleotide sequences used for gene expression quantification.

Genes	Forward (5′-3′)	Reverse (3′-5′)	Reference
*ef1α*	CTGGAGGCCAGCTCAAACAT	ATCAAGAAGAGTAGTACCGCTAGCATTAC	[38]
*rpl13α*	TCTGGAGGACTGTAAGAGGTATGC	AGACGCACAATCTTGAGAGCAG	[38]
*β-actin*	CGAGCTGTCTTCCCATCCA	TCACCAACGTAGCTGTCTTTCTG	[38]
*dmrt1*	CTGCAATGTCCAGCAGAGGGCA	GCTCAGGGCAGGTGCTTGGT	[3]
*sox9a*	TACCCGCACCTCCACAACGC	TTCTGGTGACCGTTCGGCGG	[3]
*foxl2*	GGTCGCCTGCACCCATGTCA	CGTGGCGGGACTGAGTTGCT	
*cyp19a1a*	GAGCGCAGAGAACGTCAGGCA	ACGGAGAAACGAGACAGCAG	[3]
*amh*	CAGGAGGCAGGCCGGAAGATG	CGGCATTCGCACTTGGTCGC	[3]

## Data Availability

The original contributions presented in this study are included in the article/Supplementary Materials. Further inquiries can be directed to the corresponding authors.

## Supplementary Materials

The following supporting information can be downloaded at: https://www.mdpi.com/article/10.3390/jox16030101/s1, Figure S1: qPCR validation of reference genes in zebrafish; Figure S2: qPCR validation of male-related genes in zebrafish; Figure S3: qPCR validation of female-related genes in zebrafish; Figure S4: Representative undifferentiated gonads in zebrafish (65 dpf) exposed to glyphosate and combina-tion treatments. (H&E, scale = 50 µm); Table S1: Amplification efficiencies of reference and sex determination genes used in qPCR assays; Table S2: ΔScore-based classification criteria for inferred sex; Table S3: Classification of zebrafish individuals according to inferred sex based on gene expression pro-files; Supplementary File S1: gene expression; Supplementary File S2: MDA 30dpf; Supplementary File S3: sex classification; Supplementary File S4: original images.

## Abbreviations

The following abbreviations are used in this manuscript:

GLY	Glyphosate
CPF	Chlorpyrifos
ROS	Reactive Oxygen Species
MDA	Malondialdehyde
qPCR	Quantitative Polymerase Chain Reaction
dpf	Days post fertilisation
hpf	Hours post fertilisation
RNA	Ribonucleic Acid
cDNA	Complementary DNA
PBS	Phosphate-Buffered Saline
ANOVA	Analysis of Variance
CIAD	Centro de Investigación en Alimentación y Desarrollo
EDCs	Endocrine Disrupting Chemicals
BPA	Bisphenol A
EE2	17α-ethinylestradiol
HFPO-TA	Hexafluoropropylene oxide trimer acid
TBT	Tributyltin
NAD	Nandrolone
MAPK	Mitogen-Activated Protein Kinase
