# Supplementary material for: Exposure to Glyphosate and Chlorpyrifos Induces Oxidative Stress, Potentially Impacting Sex Determination in Zebrafish (Danio rerio)
Source: J Xenobiot. 2026 Jun 2;16(3):101. doi: 10.3390/jox16030101 (PMC13301101; doi:10.3390/jox16030101)
Supplement: Supplementary file 1 [file jox-16-00101-s001.zip › Supplementary Figures and Tables.pdf]

Supplementary Materials: Exposure to Glyphosate and Chlorpyrifos Induces Oxidative Stress, Potentially Impacting Sex Determination in Zebrafish (Danio rerio)

Arias-Camacho Daniela, Rochin-Peraza Brian Antonio, Betancourt-Lozano Miguel, Abad-Rosales Selene, Heredia José Basilio, Leyva-López Nayely, Calderón-Liévanos Samuel and García-Gasca Alejandra

Figure S1 – qPCR validation reference genes
Figure S2 – qPCR validation of male genes
Figure S3 – qPCR validation of female genes
Figure S4 – Undifferentiated gonads
Table S1 – qPCR amplification efficiencies
Table S2 – Score classification criteria
Table S3 – Individual gene expression and inferred sex

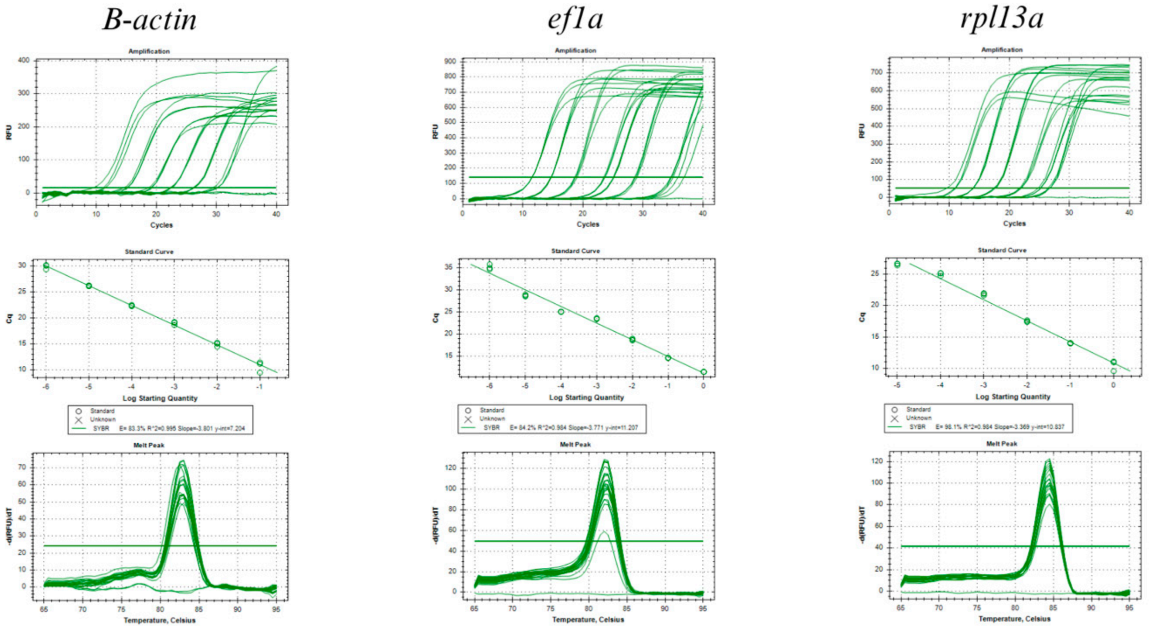

Figure S1. qPCR validation of reference genes in zebrafish.
Amplification plots, standard curves, and melt peak analysis for reference genes β-*actin*, *ef1α*, and *rpl13α* used in the study. These panels demonstrate primer specificity, amplification efficiency, and absence of non-specific products.

https://doi.org/10.3390/jox16030101

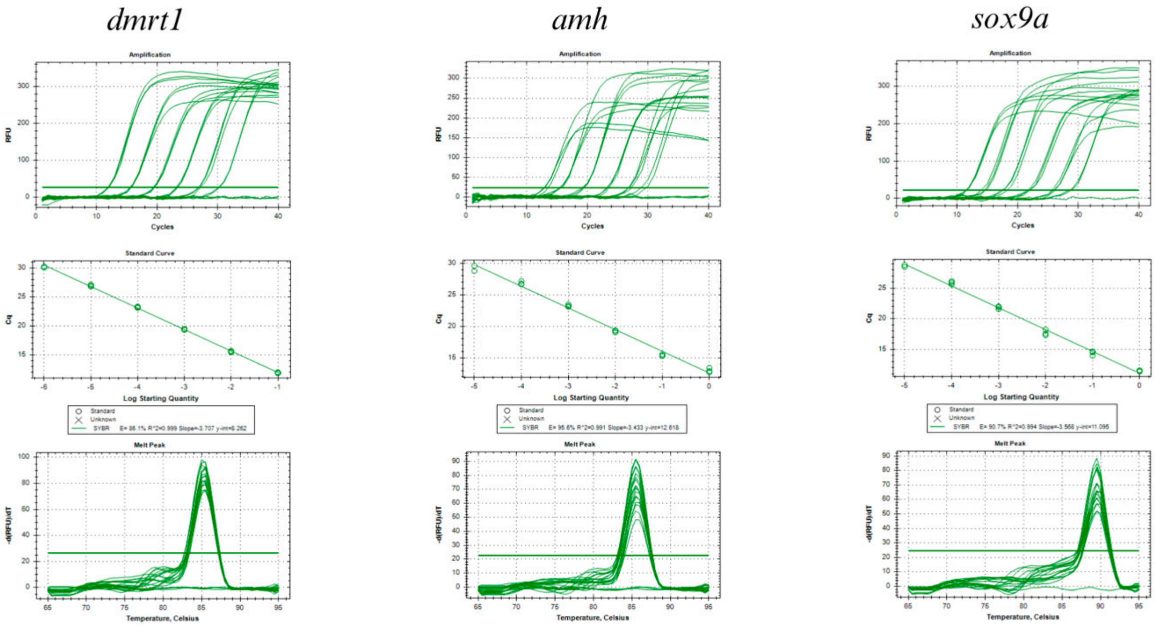

Figure S2. qPCR validation of male-related genes in zebrafish.
Amplification plots, standard curves, and melt peak analysis for male-associated genes *amh, dmrt1*, and *sox9a*. These data confirm primer specificity, amplification efficiency, and absence of non-specific products in the qPCR assays.

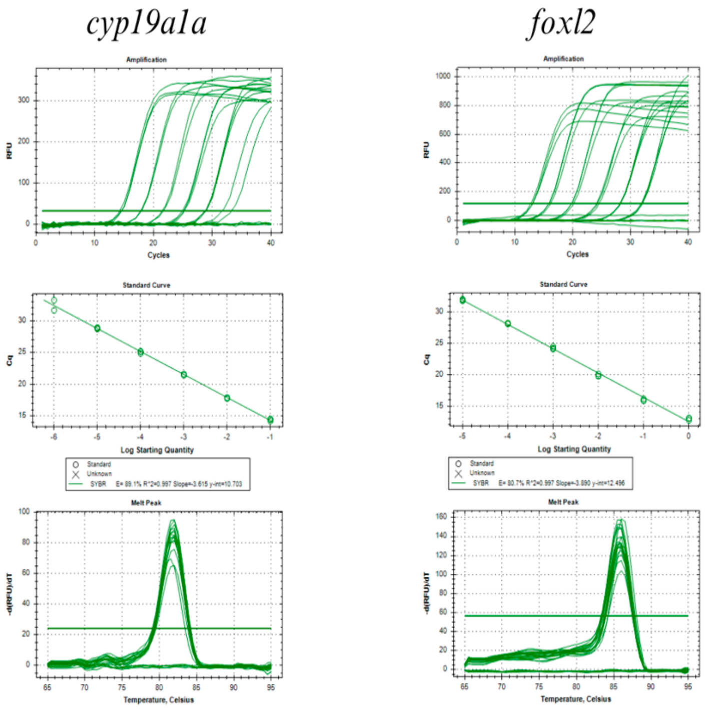

Figure S3. qPCR validation of female-related genes in zebrafish.
Amplification plots, standard curves, and melt peak analysis for female-associated genes *cyp19a1a* and *foxl2*. These data confirm primer specificity, amplification efficiency, and absence of non-specific products in the qPCR assays.

https://doi.org/10.3390/jox16030101

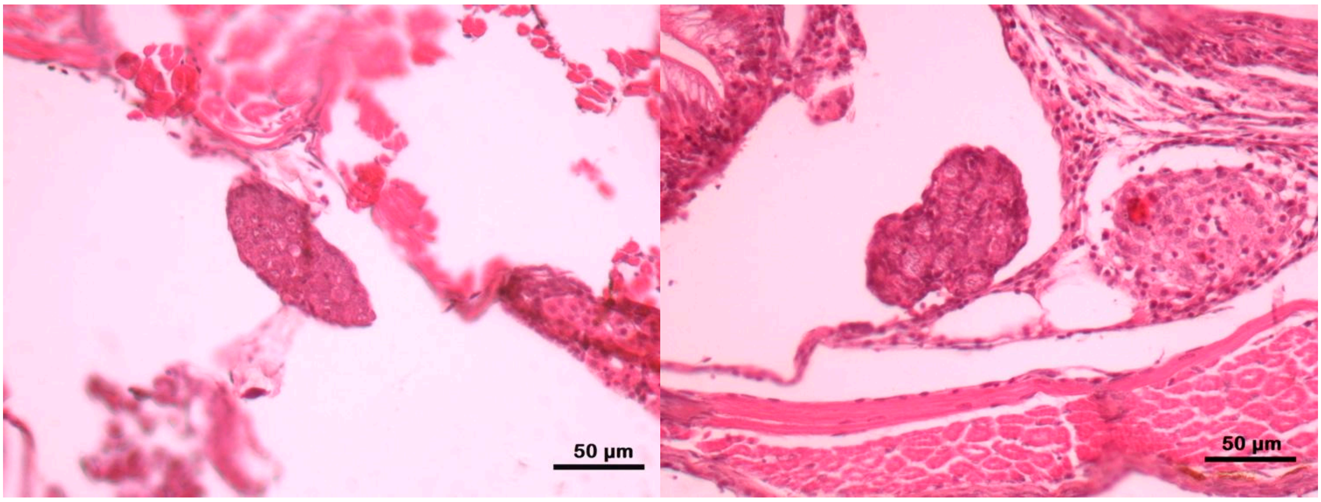

Figure S4. Representative undifferentiated gonads in zebrafish (65 dpf) exposed to glyphosate and combination treatments. (H&E, scale = 50 µm).

Table S1. Amplification efficiencies of reference and sex determination genes used in qPCR assays.

Gene	Slope	R^2	Efficiency (%)	Amplification factor
B-actina	-3.801	0.995	83.30%	1.83
ef1a	-3.771	0.984	84.20%	1.84
rpl13a	-3.369	0.984	98.10%	1.98
dmrt1	-3.707	0.999	86.10%	1.86
amh	-3.433	0.991	95.60%	1.96
sox9a	-3.568	0.994	90.70%	1.91
cyp19a1a	-3.615	0.997	89.10%	1.89
foxl2	-3.89	0.997	80.70%	1.81

Note: Amplification efficiencies (E) were calculated from the slope of the standard curve using the equation $E = 10^{-1/slope}$. Gene expression was calculated considering amplification efficiencies according to the Pfaffl (2004) method.

Table S2. ΔScore-based classification criteria for inferred sex.

**Presumed sex**	**ΔScore value**	**Description**
Male	ΔScore ≥ 1	Individuals showing predominance of male-associated gene expression over female-associated genes.
Female	ΔScore ≤ -1	Individuals showing predominance of female-associated gene expression over male-associated genes.
Intermediate (Intersex)	-1 < ΔScore < 1	Individuals in the process of sexual determination show a mixed or balanced expression pattern between male- and female-associated genes.
Indeterminate	NA / insufficient	Individuals that do not appear to have reached sexual determination; they showed no clear pattern or lacked sufficient data to assign a sex.

Note: ΔScore was calculated as ΔScore = (dmrt1 + amh + sox9a) − (cyp19a1a + foxl2). This table summarises the classification criteria applied to all individuals in Table S3.

https://doi.org/10.3390/jox16030101

Table S3. Classification of zebrafish individuals according to inferred sex based on gene expression profiles.

Sample	Treatment	*dmrt1*	*amh*	*sox9a*	*cyp19a1a*	*foxl2*	Δscore	Inferred sex
1a	Control	3.74863688	0.44936304	1.75939115	-0.4728098	NA	6.430200839	Male
1b	Control	4.04869214	3.70458867	0.82220664	-0.1511217	NA	8.726609198	Male
2a	Control	5.52783138	-4.1781471	-3.0357196	1.37549701	-0.4185446	-2.642987664	Female
2b	Control	-3.5740268	-0.3353588	3.07919259	-3.9728295	5.99305523	-2.850418704	Female
3a	Control	4.39067392	2.03227531	1.09011709	-4.6843611	NA	12.19742738	Male
3b	Control	2.48502151	-4.1265101	-1.4080063	-4.8088717	-3.2013885	4.960765299	Male
4a	Control	3.12840373	4.92043838	-1.9323391	2.04784737	1.2484707	2.820184963	Male
4b	Control	-3.9216687	-3.1188663	0.62767033	6.29275187	NA	-12.70561659	Female
5a	Control	-11.501644	NA	-0.4521113	-0.8600607	-5.5570614	-5.536633016	Female
5b	Control	-4.3319203	0.55118639	-0.5504016	5.23395822	-2.4479382	-7.117155471	Female
1a	Chlorpyrifos	-3.5948942	NA	NA	-2.9385411	-3.2375854	2.581232344	Female
1b	Chlorpyrifos	0.23032168	NA	-5.1260246	-4.2089326	NA	-0.686770334	Intersex
2a	Chlorpyrifos	-1.0271647	15.1937008	NA	-2.6196627	4.74430476	12.04189408	Male
2b	Chlorpyrifos	1.17121777	1.42714127	NA	10.3784323	NA	-7.78007323	Female
3a	Chlorpyrifos	-1.1363422	14.1976014	-2.8261412	-3.4539601	-2.5917055	16.28078353	Male
3b	Chlorpyrifos	5.01143673	7.88939109	3.57322797	-7.1793505	-0.808389	24.46179534	Male

4a	Chlorpyrifos	-1.3055613	NA	NA	-1.5085586	0.34129187	-0.138294599	Intersex
4b	Chlorpyrifos	-2.0695775	NA	NA	9.46422856	-4.0124102	-7.521395869	Female
5a	Chlorpyrifos	-4.6271618	15.4955718	NA	NA	2.51537473	8.353035283	Male
5b	Chlorpyrifos	-0.8443912	NA	NA	5.30946099	-12.222381	6.068528838	Male
1a	Glyphosate	-3.6229507	0.306489	-0.5689988	-0.0799709	2.10018247	-5.905672109	Female
1b	Glyphosate	-2.0061875	NA	-3.5205887	-5.5076755	NA	-0.019100653	Intersex
2a	Glyphosate	-2.0086633	NA	-0.8095402	NA	NA	-2.818203574	Indeterminate
2b	Glyphosate	-0.86458	NA	-1.5469023	NA	NA	-2.411482282	Indeterminate
3a	Glyphosate	-3.4634629	-0.1935432	NA	-3.1869124	-0.2741926	-0.195901007	Intersex
3b	Glyphosate	-0.4644462	NA	NA	-2.0656989	NA	1.601252704	Indeterminate
4a	Glyphosate	9.61397862	NA	3.56845682	NA	NA	13.18243544	Male
4b	Glyphosate	-6.5366668	NA	0.20309935	NA	NA	-6.333567443	Indeterminate
5a	Glyphosate	-3.3925372	0.94932074	-5.8358458	3.12664152	NA	-11.40570378	Female
5b	Glyphosate	8.02001026	13.5720105	NA	NA	NA	21.59202077	Male
1a	Combination	5.26470275	NA	-4.0994434	-3.4621038	NA	4.627363152	Male
1b	Combination	-5.6290832	10.4282143	-0.8312209	NA	NA	3.967910308	Male
2a	Combination	6.75894505	15.8506682	1.89118011	NA	7.78056451	16.72022885	Male
2b	Combination	-	9.468855	2.146681	NA	2.386287	5.3110	Male

		3.9181605	19	75		99	88495	
3a	Combination	0.81493803	13.2486192	2.06224631	3.19206233	-2.317828	15.25156916	Male
3b	Combination	-3.1676779	5.02086197	-0.9170917	2.16793593	-7.2343407	6.002497221	Male
4a	Combination	5.73367919	12.1332017	7.10786603	11.933879	7.18608817	5.854779794	Male
4b	Combination	2.39854123	21.8444596	9.97687339	4.06455746	3.75784275	26.39747398	Male
5a	Combination	3.52381716	14.768483	3.33435608	6.6360796	9.46972732	5.520849338	Male
5b	Combination	4.20386221	9.66397752	NA	NA	NA	13.86783973	Male

Note: Individuals were classified as Male, Female, Intermediate (Intersex/Undifferentiated), or Indeterminate based on the ΔScore, which integrates the relative expression of male-associated genes (*dmrt1, amh, sox9a*) and female-associated genes (*cyp19a1a, foxl2*). "NA" indicates missing data for a specific gene, which may result in an Indeterminate classification.
